# The place of EMDR in children: a review of the literature

**DOI:** 10.1192/j.eurpsy.2023.1541

**Published:** 2023-07-19

**Authors:** M. Raissouni, S. Benhammou, S. Belbachir

**Affiliations:** Arrazi de Salé, Salé

## Abstract

**Introduction:**

EMDR therapy is a brief form of trauma-focused therapy for PTSD. This therapeutic technique was first used by Francine Shapiro in the United States in 1987. EMDR combines exposure imagination, cognitive and psychoanalytical techniques. Designed to treat psychological trauma, emotional shock and grief, EMDR can also be used to treat neurotic problems such as phobias, anxiety and depression.

EMDR therapy can be used at any age and can be adapted to children, adolescents, adults and the elderly unless contraindicated.

In the pediatric population, several studies have shown that EMDR therapy has a remarkable effectiveness on several pathologies and particularly on PTSD.

Translated with www.DeepL.com/Translator (free version)

EMDR therapy is a brief form of trauma-focused therapy for PTSD. This therapeutic technique was first used by Francine Shapiro in the United States in 1987. EMDR combines exposure imagination, cognitive and psychoanalytical techniques. Designed to treat psychological trauma, emotional shock and grief, EMDR can also be used to treat neurotic problems such as phobias, anxiety and depression. EMDR therapy can be used at any age and can be adapted to children, adolescents, adults and the elderly unless contraindicated.In the pediatric population, several studies have shown that EMDR therapy has a remarkable effectiveness on several pathologies and particularly on PTSD. Translated with www.DeepL.com/Translator (free version)

**Objectives:**

Present the indications of EMDR therapy in children and demonstrated its effectiveness in the management of psychiatric disorders in this population.

**Methods:**

This is a systematic review of the literature. The databases used are “PubMed” and “Google Scholar”. No language restrictions were applied.

The following keywords were entered: EMDR, Child. Recent articles published in English or “systematic reviews, meta-analyses or reviews” were included.

**Results:**

From this review of the literature, we note that:

EMDR is a psychotherapy that has been shown to be effective in improving the symptoms of various pathologies in children, including PTSD, major depressive disorders, intellectual disability, anxiety disorders, as well as behavioral disorders

However, the studies are still few in number and also have methodological limitations: they were exploratory studies of relatively small samples.

**Conclusions:**

EMDR therapy is mentioned in the described guidelines as “promising”, as the fact that it yields positive results in a short period of time and that these results continue in follow-up studies has increased the interest in using EMDR in children.

Nevertheless, larger sample sizes are recommended in future studies to better establish the effectiveness of EMDR therapy in children.

**Disclosure of Interest:**

None Declared

